# Qidong Yixin Oral Liquid for Viral Myocarditis: A Systematic Review and Meta-Analysis

**DOI:** 10.1155/2020/4704535

**Published:** 2020-05-22

**Authors:** Jun Hu, Yu-meng Tan, Jie Wang, Hao-qiang He, Jing-yi Wang

**Affiliations:** ^1^Department of Cardiology, Guang'anmen Hospital, China Academy of Chinese Medical Sciences, Beixiange 5, Xicheng District, Beijing 100053, China; ^2^Beijing University of Chinese Medicine, Beijing 100029, China

## Abstract

**Objective:**

This study aimed to evaluate the efficacy and safety of Qidong Yixin (QY) oral liquid in the treatment of viral myocarditis (VMC).

**Methods:**

We searched seven databases for randomized clinical trials on QY for treating VMC. The retrieval period was from database establishment to December 31, 2019. Cochrane risk of bias tool in the Cochrane Handbook was used to assess the methodological quality. Review Manager (RevMan) 5.3 was used to analyze the results.

**Results:**

We included 19 studies comprising 2,608 patients, albeit with low methodological quality. Our meta-analysis revealed that combination therapy with QY and western medicine was more effective than western medicine alone (QY vs other Chinese patent medicines: RR = 1.37, 95% Cl: 1.23∼1.52, *P* < 0.00001; QY + coenzyme Q10 + routine treatment vs coenzyme Q10 + routine treatment: RR = 1.20, 95% Cl: 1.14∼1.27, *P* < 0.00001; QY + trimetazidine + acyclovir vs trimetazidine + acyclovir: RR = 1.59, 95% Cl: 1.38∼1.83, *P* < 0.00001; QY + routine treatment vs routine treatment: RR = 1.09, 95% Cl: 1.03∼1.15, *P* < 0.003). A study on posttreatment myocardial enzyme levels revealed that QY with western medicine downregulated creatine kinase isoenzyme (CK-MB) (QY + antiviral treatment + routine treatment vs antiviral treatment + routine treatment group: MD = −11.28, 95% CI: −13.33∼−9.22, *P* < 0.00001; QY + routine treatment vs routine treatment: MD = −4.96, 95% CI: −5.56∼−4.32, *P* < 0.00001), creatine kinase (CK) (MD = −32.10, 95% CI: −35.63∼−28.57, *P* < 0.00001), and lactate dehydrogenase (LDH) (QY + antiviral treatment + routine treatment vs antiviral treatment + routine treatment: MD = −48.76 95% CI: −58.18∼−39.33, *P* < 0.00001; QY + routine treatment vs routine treatment: MD = −23.52, 95% CI: −30.10–16.94, *P* < 0.00001) rather than western medicine alone, with no evidence of aspartate aminotransferase (AST) downregulation on treatment with QY with western medicine (MD = 2.88, 95% CI: −0.95∼6.71, *P* < 0.00001) in patients. Two studies reported adverse events, indicating that QY is relatively safe.

**Conclusion:**

Although QY may have potential advantages in treating VMC, they remain unclear owing to the poor methodological quality of most studies. Larger, multicenter, high-quality randomized controlled trials are required to verify the effectiveness of QY.

## 1. Introduction

According to the WHO classification of cardiomyopathy, myocarditis is defined as an inflammatory myocardial disease [1]. Viral myocarditis (VMC) is usually caused by viral myocardial infections of coxsackievirus B3, enterovirus, adenovirus, parvovirus B19, and human herpesvirus 6 [2, 3]. Myocarditis is significantly associated with mortality and is often a prominent cause of acute heart failure, severe ventricular arrhythmia, or cardiogenic shock. Myocarditis causes sudden cardiac death in up to 12% of young adults and dilated cardiomyopathy in 9% of patients [4–9]. Moreover, VMC may cause a series of severe complications and affect the long-term prognosis of patients. However, at present, myocarditis treatment methods are primarily focused on myocardial nutrition, myocardial metabolism improvement, and cardiac failure and arrhythmia treatment, while antiviral therapy and immunotherapy have not significantly benefited patients thus far [10–16].

The traditional Chinese medicine theory suggests that viral myocarditis presents as palpitations resulting from heat-toxicity invading the heart, consuming qi and injuring yin. Therefore, treatment should focus on benefiting qi and nourishing yin, thus clearing heat and eliminating toxicity. Qidong Yixin oral liquid (QY) is composed of Ginseng Radix et Rhizoma, Ophiopogonis Radix, Astragali Radix, Poria, Lonicerae japonicae flos, Epimedii Folium, Fluoritum, Testudinis Carapax et Plastrum, Rehmanniae Radix, Curcumae Radix, Cinnamomi Ramulus, Salviae Miltiorrhizae Radix et Rhizoma, and Fructus Aurantii; most of these components protect damaged cardiomyocytes [17, 18]. An animal study reported that QY activates the Nrf2/HO-1 signaling pathway, thereby reducing adriamycin-induced myocardial injury in mice [19]. Therefore, treatment of VMC with QY and combination therapy with traditional and western medicine has been common in China over the past few decades. However, most current single-center studies include small cohorts, and the treatment schemes vary greatly; hence, it is difficult to effectively evaluate the clinical efficacy of these treatment strategies. Therefore, this meta-analysis aimed to assess the efficacy and safety of combination therapeutic strategies involving QY to treat VMC, providing evidence for clinical practice.

## 2. Methods

### 2.1. Search Strategy

We searched 7 electronic databases including PubMed, Cochrane library, Embase, China National Knowledge Infrastructure (CNKI) database, Chinese scientific journal database (VIP), Wanfang database, and Chinese Biomedical Literature Service System (SinoMed). The retrieval time was from database establishment up to December 31, 2019. Reference lists from the resulting publications and reviews were used to identify further relevant publications. The retrieval formulae were as follows:

1. Chinese Biomedical Literature database (CBM)
2. Keywords: (“viral myocarditis” OR “myocarditis”) AND (“qidongyixin”)
3. CNKI
4. SU = (“viral myocarditis”+“myocarditis”) ∗ (“qidongyixin”) AND FT = (“random”)
5. Wanfang database
6. Title or key words: ((“viral myocarditis”+“myocarditis”) ∗ (“qidongyixin”)) ∗ all:(“ random”)
7. Viper database (VIP)
8. (M = (viral myocarditis OR myocarditis)) AND (M = (qidongyixin)) AND R = random
9. Medline
10. Search ((viral myocarditis [Title/Abstract]) OR myocarditis[Title/Abstract]) AND (((randomized controlled trial[Publication Type]) OR randomized[Title/Abstract]) OR placebo[Title/Abstract]) AND (qidongyixin[Title/Abstract])
11. Embase
12. #1 “viral myocarditis” OR “myocarditis”:ab, ti
13. #2 “qidongyixin”
14. #3 “Randomized” OR “placebo” OR “Randomly” OR “trial” OR “goups”[ti, ab]
15. #4 “Randomized controlled trial” OR “controlled clinical trial”[pt]
16. #5 #3 OR #4
17. #6 #1AND #2AND#5
18. Cochrane
19. #1 MeSH descriptor: [Viral Myocarditis]
20. #2 (qidongyixin):ti, ab, kw
21. #3 (randomized):ti, ab, kw
22. #4 #1 AND #2 AND #3

### 2.2. Inclusion Criteria

(1) Types of trials: Randomized controlled trials (RCTs) on QY monotherapy or combination therapy with western medicine for treating VMC were included. (2) Types of patients: patients who met the diagnostic criteria for adult VMC [20] or the diagnostic criteria for VMC formulated by the Chinese Academy of Pediatrics [21] were included irrespective of their age. (3) Types of interventional measures: the control group was treated with agents for myocardial nourishment and for improving myocardial metabolism, antiviral agents, and other routine treatments or other forms of proprietary Chinese medicine, while the experimental group was administered QY monotherapy or routine therapeutic interventions. (4) Types of outcome measures: the primary outcome indicator was total clinical efficacy [total clinical efficacy = (number of obvious cases + number of effective cases)/(total number of cases) × 100%]. Obvious cases were defined by the obliteration of most of the primary signs and symptoms after treatment and reverting of electrocardiographic findings to normalcy. Effective cases were defined as partial obliteration of the primary signs and symptoms and significant improvement in echocardiographic findings. Secondary indicators included adverse reactions and upregulation of myocardial enzymes including creatine phosphokinase (CK), creatine kinase isoenzyme (CK-MB), lactate dehydrogenase (LDH), and aspartate aminotransferase (AST).

### 2.3. Exclusion Criteria

Studies were excluded if (1) they were not randomized controlled trials and instead were retrospective studies, case reports, or reviews; (2) they included patients with severe complications; (3) they were not aimed at diagnosing VMC; (4) they contained incomplete or erroneous data; or (5) they were duplicate publications.

### 2.4. Data Extraction

Based on the PRISMA flowchart, two researchers independently screened the literature, extracted the information, evaluated the methodological quality, and cross-checked the data. Inconsistencies were discussed and negotiated with the third researcher. The data extracted herein were the following: first author, publication time, sample size, age, sex ratio, interventional measures, course of treatment, and outcome indicators.

### 2.5. Quality Assessment

Based on the criteria of the Cochrane risk of bias assessment tool, two authors independently assessed the methodological quality of the included studies, using RevMan 5.3. Disagreements were settled through discussion with a third author. The following items were evaluated: random sequence generation (selection bias), allocation concealment (selection bias), blinding of participants and personnel (performance bias), blinding of outcome assessment (detection bias), incomplete outcome data (attrition bias), and selective outcome reporting (reporting bias). Other potential sources of bias included sample size estimates and the comparability of baseline characteristics. Each included randomized controlled trial was classified as being of a low, ambiguous, or high risk of bias for quality assessment. Studies that met all criteria were classified as being of a high risk of bias, whereas those that did not meet any criteria were classified as being of a low risk of bias. Others were classified as being of an ambiguous risk of bias.

### 2.6. Data Synthesis and Analysis

RevMan 5.3 was used for the meta-analysis of multiple studies. Continuous data are expressed as weighted average difference (WMD) values, and dichotomous data are expressed as relative risk (RR), both using a 95% confidence interval (CI). The heterogeneity of the study was qualitatively evaluated by the Q test and quantitatively evaluated by the *I*^2^ test. When there was no significant heterogeneity among multiple studies (*P* ≥ 0.10, *I*^2^ ≤ 50%), we used the fixed effects model to analyze the data. If there was substantial heterogeneity (*P* < 0.10, *I*^2^ > 50%), a random effects model was established and the possible sources of heterogeneity were investigated using sensitivity analysis and subgroup analysis. When more than 10 trials were included, funnel plots were generated to detect publication bias.

## 3. Results

### 3.1. Study Search and Selection

**Figure 1:**
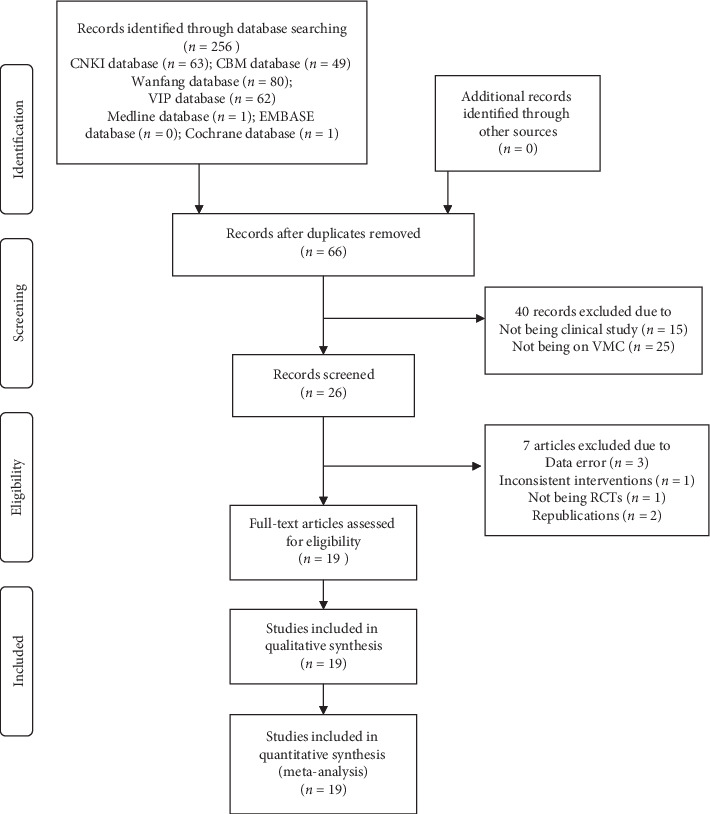
Flow diagram of literature retrieval.

We initially retrieved 256 studies on VMC treatment from 7 databases. After eliminating 190 duplicate publications, 66 articles were obtained. After reviewing the titles and abstracts of these studies, we excluded 40 articles, of which 25 were on nonviral myocarditis, 15 were nonclinical studies, and the remaining 26 were clinical studies. After full-text review, significant errors in the data from three studies, two duplicate studies, inconsistent interventions in one study, and nonrandomized controlled trials in one study were noted. After excluding these studies, 19 studies remained [22–40]. The filtering process is shown in Figure 1.

### 3.2. Characteristics of the Included Studies

The characteristics of the 19 RCTs are shown in Table 1. These RCTs included 2,608 patients, nine of which included children [24, 26, 28, 30, 32, 33, 37, 38, 40], nine included adults [22–25, 27, 29, 31, 36], and one involved elderly patients [39]. These 19 RCTs were published between 1996 and 2019, and all included patients met the diagnostic criteria for viral myocarditis. Two RCTs compared the efficacy of QY with that of other proprietary Chinese medicines [35, 36]. Eight RCTs compared the efficacy of combination therapy with QY + coenzyme Q10 + routine treatment with that of coenzyme Q10 + routine treatment [22, 24, 25, 27, 30, 37, 39, 40]. Four RCTs compared the efficacy of QY + trimetazidine + acyclovir with that of trimetazidine + acyclovir [23, 32–34]. Four RCTs compared the efficacy of QY + routine treatment with that of routine treatment alone [26, 28, 31, 38]. Four RCTs involved antiviral therapy [24, 26, 33, 38]. In 10 RCTs, the daily QY dose was 60 ml [22, 23, 27, 29, 31–34, 36, 39], in 3 RCTs of which, patients received a daily QY dose of 30 ml [25, 35, 37], and in the other 6, patients received different doses (based on age and weight) [24, 26, 28, 30, 38, 40]. The course of treatment was 1–12 weeks. In terms of outcome indicators, eighteen RCTs reported the total clinical efficacy [22–28, 30–40], 7 RCTs reported changes in CK-MB levels after treatment [24, 26, 29–31, 33, 38], 5 RCTs reported changes in CK levels after treatment [24, 26, 29, 37, 38], 5 RCTs reported changes in LDH levels after treatment [24, 29, 33, 37, 38], and only 3 RCTs reported changes in AST levels after treatment [29, 37, 38]. Adverse events were reported in 2 RCTs [22, 24].

### 3.3. Risk of Bias in the Included Studies/Methodological Quality

**Figure 2:**
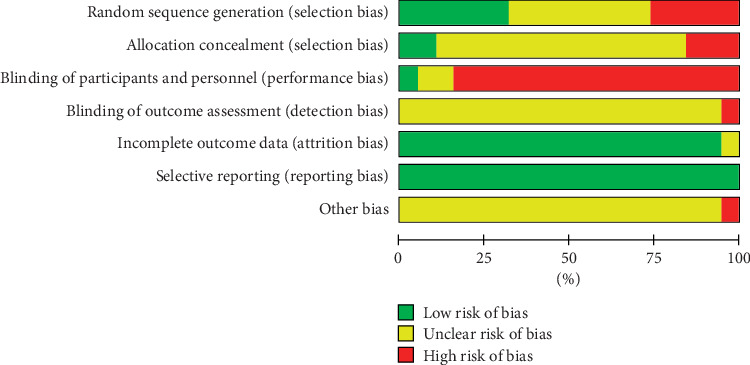
Risk of bias summary.

**Figure 3:**
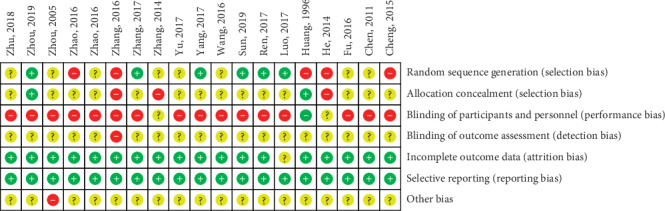
Risk of bias graph.

Among 19 RCTs, 7 used the random number table method [26–31, 39], 2 adopted the odd-even method [24, 34], 5 included the term “randomization” but did not elaborate on the randomization method [22, 32, 33, 37, 38], and 4 did not include the term “randomization” [23, 35, 36, 40]. Furthermore, 1 study was double-blinded [36], while the others were not. All trials had a high or unclear risk of bias (Figure 2 and Figure 3).

### 3.4. Effects of the Interventions

#### 3.4.1. Clinical Efficacy

**Figure 4:**
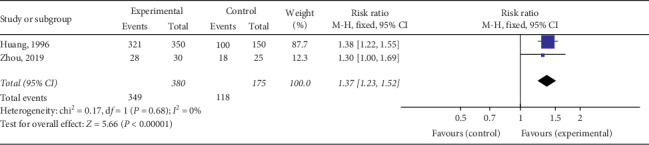
Forest plot of the comparison between QY and other Chinese patent medicines for total clinical efficacy.

**Figure 5:**
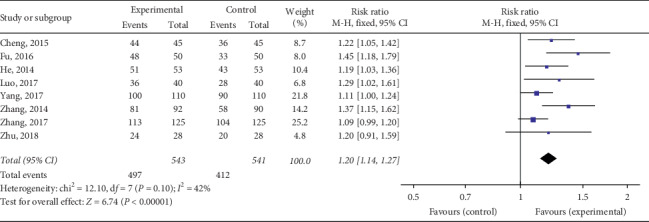
Forest plot of the comparison between QY + coenzyme Q10 + routine treatment and coenzyme Q10 + routine treatment for total clinical efficacy.

**Figure 6:**
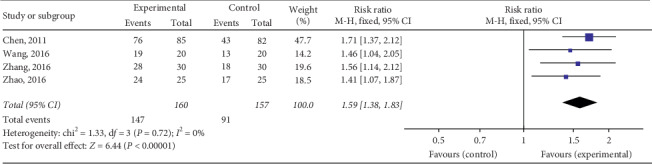
Forest plot of the comparison between QY + trimetazidine + acyclovir and trimetazidine + acyclovir for total clinical efficacy.

**Figure 7:**
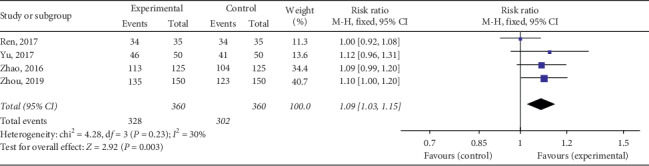
Forest plot of the comparison between QY + routine treatment and routine treatment alone for total clinical efficacy.

The total effective rate of QY in treating VMC was reported in 18 RCTs including 2090 cases in total. Considering various treatment methods for VMC in each study, we divided them into four subgroups based on different treatment methods to ensure the comparability of various studies. The meta-analysis revealed minor heterogeneity in each subgroup. The fixed effects model was used for the combined analysis, and the total effective rate of the test group was higher than that of the control group, among which the total effective rate of QY was compared to that of other Chinese patent medicines [Chi^2^ = 0.17, d*f* = 1(*P*=0.68), *I*^2^ = 0%; RR = 1.37, 95% Cl: 1.23∼1.52, *P* < 0.00001] (Figure 4). QY + coenzyme Q10 + routine treatment was compared with coenzyme Q10 + routine treatment [Chi^2^ = 12.10, d*f* = 7(*P*=0.10), *I*^2^ = 42%; RR = 1.20, 95% Cl: 1.14∼1.27, *P* < 0.00001] (Figure 5). QY + trimetazidine + acyclovir treatment was compared with trimetazidine + acyclovir treatment [Chi^2^ = 1.33, d*f* = 3 (*P*=0.72), *I*^2^ = 0%; RR = 1.59, 95% Cl: 1.38∼1.83, *P* < 0.00001] (Figure 6). QY + routine treatment was compared with routine treatment alone [Chi^2^ = 4.28, d*f* = 3 (*P*=0.23), *I*^2^ = 30%; RR = 1.09, 95% Cl: 1.03∼1.15, *P* < 0.003] (Figure 7). These results indicate that QY significantly improved the clinical total effective rate among VMC patients.

**Figure 8:**
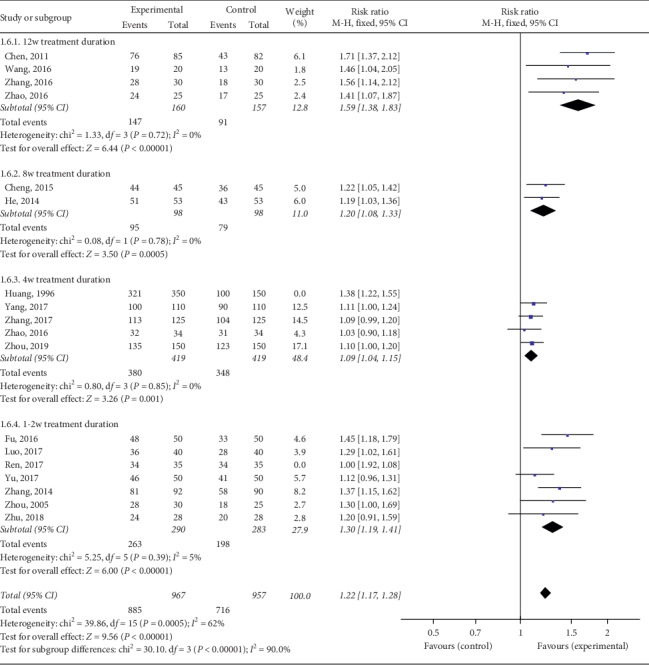
Forest plots of subgroups analyzed by course of treatment.

We also divided the study into four subgroups according to different courses of treatment. The total effective rate of the experimental group was higher than that of the control group, among which the 12 w treatment duration group [Chi^2^ = 1.33, d*f* = 3 (*P*=0.72), *I*^2^ = 0%; RR = 1.59, 95% Cl: 1.38∼1.83, *P* < 0.00001] and 8w treatment duration group [Chi^2^ = 0.08, d*f* = 1 (*P*=0.78), *I*^2^ = 0%; RR = 1.20, 95% Cl: 1.08∼1.33, *P*=0.0005] showed low heterogeneity. Heterogeneity was observed between the 4 w treatment duration group (*P*=0.003, *I*^2^ = 75%) and the 1-2 w treatment duration group (*P* < 0.00001, *I*^2^ = 83%). Sensitivity analysis showed that heterogeneity decreased after the removal of Huang's study from the 4 w treatment duration group [Chi^2^ = 0.80, d*f* = 3 (*P*=0.85), *I*^2^ = 0%; RR = 1.09, 95% Cl: 1.04∼1.15, *P*=0.001], probably because the control group in Huang YS used a Chinese patent medicine, which was different from that in the other studies. Heterogeneity was reduced after the removal of Ren MY from the 1-2 w treatment duration group [Chi^2^ = 5.25, d*f* = 5 (*P*=0.39), *I*^2^ = 5%; RR = 1.30, 95% Cl: 1.19∼1.41, *P* < 0.00001], which may have been caused by the low quality of research in Ren MY (Figure 8).

#### 3.4.2. CK-MB Levels

**Figure 9:**
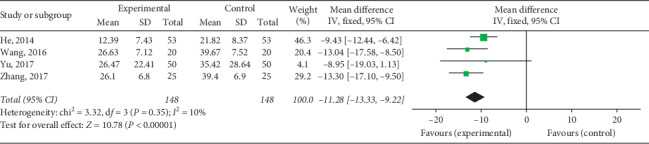
Forest plot of the comparison between QY + antiviral treatment + routine treatment and antiviral treatment + routine treatment for CK-MB.

**Figure 10:**
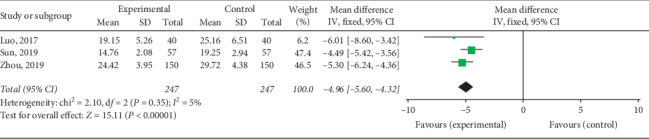
Forest plot of the comparison between QY + routine treatment and routine treatment alone for CK-MB.

CK-MB levels upon QY treatment among VMC patients were reported in 7 studies including 790 patients. Two subgroups were formed depending on whether antiviral therapy was combined. The meta-analysis revealed that the heterogeneity of each subgroup was small, and the fixed effects model was used for combined analysis. CK-MB levels in the experimental group after treatment were lower than those in the control group on comparing QY + antiviral treatment and antiviral treatment [Chi^2^ = 3.32, d*f* = 3 (*P*=0.35), *I*^2^ = 10%; MD = -11.28, 95% CI: −13.33∼−9.22, *P* < 0.00001] (Figure 9) and on comparing QY + routine treatment and routine treatment [Chi^2^ = 2.10, d*f* = 2 (*P*=0.35), *I*^2^ = 5%; MD = −4.96, 95% CI: −5.56∼−4.32, *P* < 0.00001] (Figure 10). These results indicate that QY may reduce CK-MB levels in VMC patients.

#### 3.4.3. CK Levels

**Figure 11:**
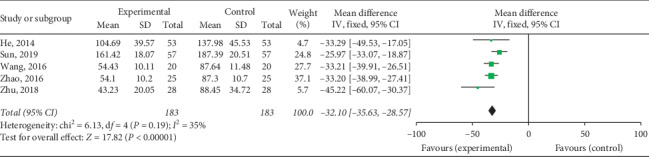
Forest plot of the comparison between QY plus western medicine and western medicine alone for CK.

Five studies including 366 patients reported changes in CK levels. The heterogeneity test and evaluation of the included studies revealed that the heterogeneity among the studies was small [Chi^2^ = 6.13, d*f* = 4 (*P*=0.68), *I*^2^ = 35%]. The fixed effects model was adopted for combination analysis, and it was found that the CK level of the experimental group after treatment was lower than that of the control group (MD = −32.10, 95% CI: −35.63∼−28.57, *P* < 0.00001) (Figure 11). These results indicate that QY reduces CK levels in VMC patients.

#### 3.4.4. LDH Levels

**Figure 12:**
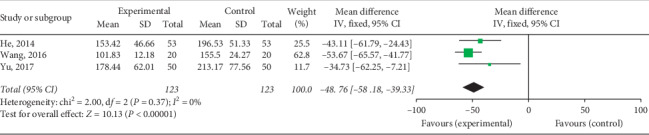
Forest plot of the comparison between QY + antiviral treatment + routine treatment and antiviral treatment + routine treatment for LDH.

**Figure 13:**
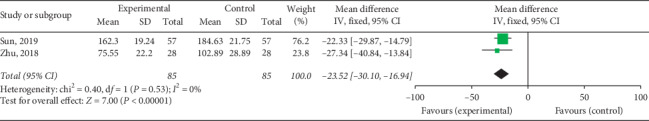
Forest plot of the comparison between QY + routine treatment and routine treatment alone for LDH.

Five RCTs including 416 patients reported changes in LDH levels after QY treatment. Two subgroups were formed depending on whether antiviral therapy was combined. The results show that the heterogeneity of each subgroup was small. The fixed effects model was used for analysis, and it was found that the LDH level of the experimental group after treatment was lower than that of the control group on comparing QY combined with antiviral treatment [Chi^2^ = 2.00, d*f* = 2(*P*=0.37), *I*^2^ = 0%; MD = −48.76 95% CI: −58.18∼−39.33, *P* < 0.00001] (Figure 12) and QY monotherapy [Chi^2^ = 0.40, d*f* = 1 (*P*=0.53), *I*^2^ = 0%; MD = −23.52, 95% CI: −30.10–16.94, *P* < 0.00001] (Figure 13), indicating that QY can reduce LDH levels in VMC patients.

#### 3.4.5. AST Levels

**Figure 14:**
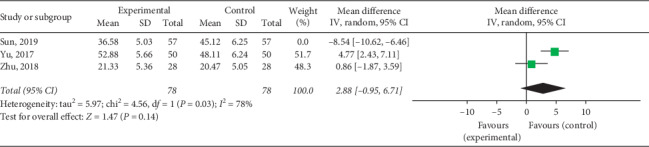
Forest plot of the comparison between QY + routine treatment and routine treatment alone for AST.

Three RCTs including 270 patients reported changes in AST levels. The heterogeneity test for the included studies revealed that the heterogeneity among the studies was large (*P* < 0.00001, *I*^2^ = 97%), and sensitivity analysis did not affect the outcomes, probably owing to noncomparability of the baseline AST levels among the included studies. Furthermore, the subjects in Sun's study [29] were adults and those in the other two studies were children. Heterogeneity was reduced upon excluding Sun's study [Chi^2^ = 4.56, d*f* = 1 (*P*=0.03), *I*^2^ = 78%]. The random effects model was used for combined analysis, and it was found that the AST level in the experimental group after treatment was higher than that in the control group (MD = 2.88, 95% CI: −0.95∼6.71, *P* < 0.00001) (Figure 14). These results indicate that QY may not have the advantage of reducing the AST level in VMC patients, and more high-quality studies are needed to increase the credibility of these results.

### 3.5. Publication Bias

We could not conduct funnel plot analysis to detect a publication bias owing to the insufficient number of experiments.

### 3.6. Adverse Effects

Two studies reporting adverse events included 288 patients, with 145 in the treatment group and 143 in the control group. In the treatment group, 2 patients had a low fever and 1 patient had a rash; however, no other adverse reactions were observed. These results indicate the safety of using QY to treat VMC.

## 4. Discussion

QY is a patented drug approved by the State Food and Drug Administration of China. This study evaluated the efficacy and safety of Qidong Yixin (QY) oral liquid for treating VMC. The present results show that QY may be an effective and safe alternative for treating VMC. The present meta-analysis revealed that combination treatment with QY and western medicine could relieve the symptoms of VMC more effectively than western medicine alone and could reduce the levels of myocardial enzymes. However, QY did not appear to have the advantage of reducing AST levels in VMC patients. Traditional Chinese medicine is a holistic medical system with unique theories and methods with prominent advantages in alleviating symptoms. QY has a high potential to improve clinical symptoms in comparison with other therapeutic methods including myocardial nourishment, improvement of myocardial metabolism, and antiviral treatment.

Furthermore, current studies have confirmed the efficacy of QY for treating VMC, and its mechanism may be associated with the improvement of immune function, improvement of serum inflammatory factor levels, reduction of myocardial injury, reduction of myocardial enzyme levels, and improvement of cardiac function. One study reported that Qidong Yixin oral solution significantly reduces myocardial injury in SD rats infected with the coxsackievirus B3, maintains consistency in spontaneous beating of myocardial cells, maintains high pulse frequency, and reduces LDH and AST release [41]. Another animal study using a BALB/c mouse model of VMC intraperitoneally administered coxsackievirus B3, divided into a blank control group, QY-treated group (at different doses), and ribavirin-treated group, reported that serum LDH levels of QY-treated mice were significantly lower than those of control mice, and the viral load decreased and antibodies were detected in this group in comparison with the control group [42].

Furthermore. Some of the included studies reported the immunoregulatory effect of QY. In one study, QY treatment significantly improved cellular immune function and levels of inflammatory factors, significantly increased the levels of cluster of differentiation 4 (CD4) and CD8 in the peripheral blood (*P* < 0.05), and significantly decreased the levels of tumor necrosis factor-alpha (TNF-*α*), interleukin-6 (IL-6), and IL-8 in comparison with basic treatment (*P* < 0.05) [25]. Another study reported that QY combined with basic treatment significantly improved cardiac function in comparison with basic therapy alone [38]. However, such reports are still rare, and more pharmacological and clinical studies are required to verify the mechanism of action of QY for treating VMC.

## 5. Strengths and Limitations

The specific limitations of this study primarily include the following: The diagnostic criteria of this study are not uniform. The included subjects had a large age range. Baseline levels were not comparable in some studies. No follow-up data are available regarding short-term curative effects. Few, irregular studies reported on the safety of the treatment. The dosage and usage of drugs in the control group were not clearly described in some studies.

## 6. Conclusions

In conclusion, combination therapy with QY and western medicine has higher efficacy and safety against VMC than western medicine alone. However, owing to the high heterogeneity, small sample size, low methodological quality, and low credibility of our results, future, more conclusive, multicenter RCTs with a high methodological quality are required to validate the present results.

## Figures and Tables

**Table 1:** Characteristics of the included studies.

Study ID first author, year	Sample size (T/C)	Age (years)	Sex (male/female)	Intervention		Treatment duration	Outcome measure
				E	C		
Zhang, 2011 [22]	182 (92/90)	T: 36.9 ± 6.2 C: 38.5 ± 5.8	99/182	QY 20 ml tid + interferon *α*-2b 1 million unit qd + coenzyme Q10 + routine treatment	Coenzyme Q10 + routine treatment	2 w	Total clinical efficacy, CK-MB, adverse reactions
Zhang, 2016 [23]	60 (30/30)	54.2 ± 2.3	33/60	QY 20 ml tid + trimetazidine 20 mg tid + acyclovir 0.1 g tid + routine treatment	Trimetazidine 20 mg tid + acyclovir 0.1 g tid + routine treatment	12 w	Total clinical efficacy
He, 2014 [24]	106 (53/53)	5.32	66/106	QY was given, 6∼20 ml tid according to age + coenzyme Q10 20 mg bid + routine treatment + antiviral treatment	Coenzyme Q10 20 mg bid + routine treatment + antiviral treatment	8 w	Total clinical efficacy, CK-MB, CK, LDH, adverse reactions
Fu, 2016 [25]	100 (50/50)	T: 33.9 ± 12.5 C: 31.7 ± 10.3	47/100	QY 15 ml bid + routine treatment	Routine treatment	15 d	Total clinical efficacy
Zhao, 2016 [26]	68 (34/34)	5.2 ± 1.7/5.8 ± 1.5	35/68	QY 5∼10 ml tid + routine treatment + antiviral treatment	Routine treatment + antiviral treatment	4 w	Total clinical efficacy
Yang, 2017 [27]	220 (110/110)	43.8 ± 12.1	114/220	QY 20 ml tid + coenzyme Q10 30–60 mg/d + routine treatment	Coenzyme Q10 30–60 mg/d + routine treatment	4 w	Total clinical efficacy
Ren, 2017 [28]	70 (35/35)	5.0 ± 1.9/5.3 ± 1.6	34/70	QY 5∼10 ml tid + routine treatment	Routine treatment	2 w	Total clinical efficacy
Sun, 2018 [29]	114 (57/57)	34.16 ± 6.24/33.67 ± 6.18	52/114	QY 20 ml tid + routine treatment	Routine treatment	2 w	CK-MB, CK, LDH, AST
Luo, 2017 [30]	80 (40/40)	5.61 ± 1.27/5.17 ± 1.33	48/80	QY was given, 10∼20 ml tid according to age + coenzyme Q10 + routine treatment	Coenzyme Q10 + routine treatment	2 w	Total clinical efficacy, CK-MB,
Zhou, 2019 [31]	300 (150/150)	47.2 ± 15.1	165/300	QY 20 ml tid + routine treatment	Routine treatment	4 w	Total clinical efficacy, CK-MB,
Chen, 2011 [32]	167 (85/82)	36.1 ± 18.1/35.8 ± 16.3	Not reported	QY 20 ml tid + trimetazidine 20 mg tid + acyclovir + routine treatment	Trimetazidine 20 mg tid + acyclovir + routine treatment	12 w	Total clinical efficacy
Wang, 2016 [33]	40 (20/20)	5.34 ± 0.29	23/40	QY 20 ml tid + trimetazidine + acyclovir + routine treatment	Trimetazidine + acyclovir + outine treatment + antiviral treatment	12 w	Total clinical efficacy, CK-MB, CK, LDH, AST
Zhao, 2016 [34]	50 (25/25)	52.9 ± 7.5/52.8 ± 7.4	26/50	QY 20 m tid + trimetazidine + acyclovir + routine treatment	Trimetazidine + acyclovir + routine treatment	12 w	Total clinical efficacy, CK-MB, CK
Zhou, 2005 [35]	55 (30/25)	31.2 ± 3.1/32.1 ± 5.1	23/55	QY 10 ml tid	Shenmai injection 50 ml qd	15 d	Total clinical efficacy
Huang, 1996 [36]	500 (350/150)	23.9 (control group not reported)	Not reported	QY 20 ml tid	Shengmaiyin oral liquid 20 ml tid	4 w	Total clinical efficacy
Zhu, 2018 [37]	56 (28/28)	8.2 ± 3.6	32/56	QY 10 ml tid + coenzyme Q10 + routine treatment	Coenzyme Q10 + routine treatment	7 d	Total clinical efficacy, CK, LDH, AST
Yu, 2017 [38]	100 (50/50)	Not reported	Not reported	QY was given, 5∼20 ml tid according to weight + routine treatment + antiviral treatment	Routine treatment + antiviral treatment	2 w	Total clinical efficacy, CK-MB, LDH, AST
Zhang, 2017 [39]	250 (125/125)	74.4 ± 6.2/73.1 ± 7.6	121/250	QY 20 ml tid + coenzyme Q10 30∼60 mg/d + routine treatment + antiarrhythmic treatment	Coenzyme Q10 30∼60 mg/d + routine treatment + antiarrhythmic treatment	4 w	Total clinical efficacy
Cheng, 2015 [40]	90 (45/45)	5.87/2.15/6.05 ± 2.24	54/90	QY was given, 6∼20 ml tid according to age + routine treatment	Coenzyme Q10 40 mg bid + routine treatment	8 w	Total clinical efficacy
